# Breastfeeding Practice in Zhejiang Province, PR China, in the Context of Melamine-contaminated Formula Milk

**DOI:** 10.3329/jhpn.v28i2.4891

**Published:** 2010-04

**Authors:** Liqian Qiu, Colin W. Binns, Yun Zhao, Andy H. Lee, Xing Xie

**Affiliations:** ^1^ School of Public Health, Curtin Health Innovation Research Institute, Curtin University of Technology, Perth, WA, Australia; ^2^ Women's Hospital, School of Medicine, Zhejiang University, Hangzhou, China

**Keywords:** Breastfeeding, Cohort studies, Disasters, Exclusive breastfeeding, Melamine, Melamine disasters, Prospective studies, China

## Abstract

A prospective cohort study of 1,520 mothers from Zhejiang province of China was undertaken to determine the duration of breastfeeding and associated factors during the first six months postpartum. Most (95.3%) mothers had introduced complementary foods by six months, making them at risk from contaminated infant formula. The mean duration of ‘any breastfeeding’ was greater than 180 days but only 48 days for ‘exclusive breastfeeding’. Factors relating to cessation of any breastfeeding were maternal age, timing of the breastfeeding decision, admission of the infant to a special-care nursery, return of the mother to work, the early introduction of water and/or other complementary foods, and their location of residence. The introduction of infant formula before three months was positively associated with the late decision to breastfeed, births in city, and infants being given a prelacteal feed. To combat the melamine disaster, strategies to improve the duration of breastfeeding must be developed taking these factors into account.

## INTRODUCTION

A recent market report concluded that the production of infant formula in the world was 907,000 tonnes during 2008, and the market is still growing rapidly. Sales were valued at US$ 9 billion, and 53% of the market was in Asia (1). China is the largest market for infant formula in the world, a market shared by the major companies for multi-national formula and about 150 local producers.

The recent contamination of infant formula with melamine in China has caused a public-health crisis, with reports of at least 50,000 infants admitted to hospitals with renal stones (2). Melamine has high nitrogen content and is added to milk to conceal the low protein content. Little information is available on the toxicity of melamine in humans but it is known to cause renal calculi, renal failure, and haematuria (2, 3). Immediately following notification of the contamination, mothers of children aged less than three years were asked to contact their local child-health clinic. In total, 1.5 million infants were screened in Zhejiang province for the use of formula brands that had been contaminated while 3,000 infants were admitted to hospitals for further assessment.

Internationally, exclusive breastfeeding is recommended until six months of age and then continued breastfeeding for 12 months and beyond (4–7). Breastfeeding has been emphasized in public-health programmes by the Chinese Ministry of Health, particularly over the past two decades. It was the centre piece of the “National Program of Action for Child Development in China in the 1990's” issued in 1994 (8). In traditional Chinese society, breastfeeding was always the focus of child health. However, despite recent increases, breastfeeding rates have still not reached the national targets.

Identifying the subgroup of mothers with earlier introduction of formula and shorter duration of breastfeeding is an important public-health strategy because they are susceptible to contaminated infant formula. Results of studies in China and Viet Nam suggest that successful breastfeeding depends on many factors relating to the mother, the infant, and the surrounding environment. The duration of breastfeeding has been shown to be positively associated with maternal age, maternal education, the hospital environment, timing of the decision to breastfeed, early use of pacifier, and time lapse before the first breastfeeding (9–13). The use of complementary feeds during hospital stay, maternal and paternal smoking, maternal obesity, and an early return to work shorten the duration of breastfeeding (14, 15).

In Zhejiang province of China, the rural areas have not progressed as rapidly as the cities and urban areas while people from the rural west and nearby provinces continue to migrate to the major cities in search of more lucrative employment. In 2006, the population in Zhejiang was 49 million. A cross-sectional survey undertaken in five cities of Zhejiang in 1997 found that the rate of exclusive breastfeeding before discharge was 74.4% but dropped to 43.7% by 10 weeks (16). This rate was lower than the target of the World Health Organization (WHO) for initiation and exclusive breastfeeding of six months. The purpose of this study was to document the breastfeeding rates and to identify the factors determining the duration of breastfeeding and the earlier introduction of infant formula in Zhejiang province. The findings are important for avoiding another melamine disaster in the future.

## MATERIALS AND METHODS

### Study sample and site

A prospective cohort study of breastfeeding was undertaken in Zhejiang province from October 2004 to December 2005. Participants were interviewed before discharge from hospital and at one, three, and six months postpartum. Mothers were recruited from Hangzhou (capital city of Zhejiang), a suburban area and a mountainous rural area, 300 km to the southwest. Of 1,551 mothers invited to participate, 1,520 (98%) agreed and consented to the study. Three hospitals were selected to be representative of the healthcare facilities of city, suburban and rural areas. Random selection of mothers was adopted in the larger facilities where a number of deliveries occurred on a particular day. The inclusion criteria for the study were: delivery of a live child; both mother and neonate did not have any serious illness; and the mother was a resident within the service area of the hospital.

### Collection of data

The questionnaire solicited information on infant-feeding methods and variables likely to affect the duration of breastfeeding. It was based on validated and reliable instruments that have been extensively used in cohort studies on breastfeeding in China, Australia, Viet Nam, and Kenya (17–21). The questionnaire was translated into Mandarin and back-translated by professionals. Its appropriateness and cultural relevance were confirmed via focus groups. All interviews were conducted by staff familiar with the local Zhejiang dialect.

### Definitions

The definitions used in a previous study (11) were adopted as follows:

- *Any breastfeeding*: The child has received breastmilk (direct from the breast or expressed) with or without other drinks, formula, or infant food.
- *Exclusive breastfeeding*: Breastfeeding while giving no other food or liquid, not even water, with the exception of drops of syrups consisting of vitamins, mineral supplements, or medicine.
- *Full breastfeeding*: Almost exclusive breastfeeding with only small amounts of other fluids.

### Analysis of data

Data were entered and analyzed using the SPSS software (version 14.0) (SPSS Inc., Chicago, IL, USA). Survival analysis was performed on those women who still breastfed at the time of discharge. The duration of breastfeeding was estimated by the Kaplan-Meier method and compared between groups using the log-rank test. Cox proportional hazards regression was then applied to determine the factors affecting the duration of ‘any’ and ‘exclusive’ breastfeeding, with significant variables selected based on the backward elimination procedure. Logistic regression analysis was conducted to identify the factors associated with the earlier introduction of infant formula.

### Ethics

Approval of ethics was obtained from the Human Research Ethics Committee of Curtin University and the participating hospitals in China. All participants were assured of confidentiality and their right to withdraw at any time. Data were subsequently de-identified to ensure privacy of the information collected.

## RESULTS

Demographic characteristics of the sample, together with the percentage of mothers still breastfeeding at six months, are shown in Table 1. The use of prelacteal formula feeds was common, with the prevalence of 62.0%, 36.6%, and 39% in the city, suburban and rural areas respectively (22). The any breastfeeding rate at discharge from hospital was 96.9% overall but dropped to 74% after six months—similar in all the three locations.

**Table 1.** Demographic factors affecting duration of any breastfeeding at six months postpartum in Zhejiang province

Factor	No.	%	95% CI	p value
Maternal education (years)				<0.001
≤9	539	84	80.1–87.9	
10–12	370	71	65.1–76.9	
≥13	595	67	63.1–70.9	
Maternal profession				0.002
Labour	490	81	77.1–84.9	
Office work	758	69	65.1–72.9	
Not working	222	76	70.1–81.9	
Maternal age (years)				0.231
≤24	355	77	71.1–82.9	
25–29	800	72	68.1–75.9	
≥30	335	78	72.1–83.9	
Maternal salary (Yuan)				<0.001
≤3,000	609	83	79.1–86.9	
3,000–5,000	452	73	67.1–78.9	
≥5,001	406	61	55.1–66.9	
Gestation (weeks)				<0.001
<37	48	47	29.4–64.6	
≥37	1,438	76	74.0–78.0	
Birthweight (g)				0.443
≤2,499	27	73	55.4–90.6	
2,500–3,999	1,377	75	73.0–77.0	
≥4,000	96	74	64.2–83.8	
Parity				0.018
Primiparous	1,341	74	72.0–76.0	
Multiparous	162	85	79.1–90.9	
Delivery method				0.268
Vaginal	492	76	72.1–79.9	
Caesarean	1,015	74	70.1–77.9	
Breastfeeding decision made				<0.001
Before pregnancy	1,087	78	74.1–81.9	
During pregnancy	260	70	64.1–75.9	
After birth	152	65	57.2–72.8	
Maternal grandmother breastfed				0.312
Yes	1,398	75	73.0–77.0	
No	80	63	49.3–76.7	
Infant admitted to special-care nursery				<0.001
Yes	149	59	49.2–68.8	
No	1,324	76	74.0–78.0	
Suck time (minutes)				0.027
≤30	480	79	75.1–82.9	
>30	960	75	71.1–78.9	
Mother attended antenatal classes				0.937
Yes	949	73	69.1–76.9	
No	549	77	73.1–80.9	
First feed				<0.001
Breastmilk	927	77	73.1–80.9	
Other	551	71	67.1–74.9	
Baby's gender				0.800
Male	769	74	70.1–77.9	
Female	728	75	71.1–78.9	
Location				<0.001
City	638	63	57.1–68.9	
Suburban	346	77	71.1–82.9	
Rural	524	84	80.1–87.9	
Back to work				<0.001
Before 6 months	497	64	60.1–67.9	
After 6 months	771	81	77.1–84.9	
Consume water				<0.001
Within 1 month	538	65	59.1–70.9	
After 1 month	947	80	78.0–82.0	
Introduction of complementary food				0.003
Within 3 months	439	69	65.1–72.9	
After 3 months	853	77	73.1–80.9	

CI=Confidence interval

**Fig. 1.**
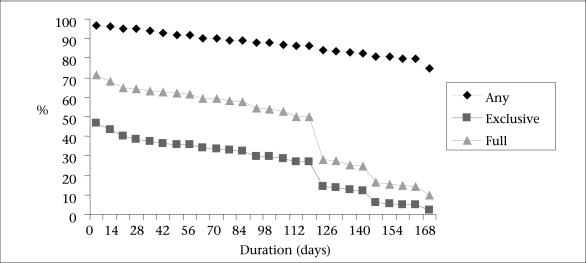
Duration of breastfeeding (any, full, and exclusive) in Zhejiang province, China, 2004–2005

Table 2 shows the details of exclusive breastfeeding in relation to the demographic and other factors. The rates of exclusive breastfeeding on discharge—38% in city, 63.4% in suburban, and 61% in rural areas—differed significantly among the three locations. The durations of exclusive, full and any breastfeeding in Zhejiang province are presented in Figure 1. The mean ages for the introduction of infant formula were 3.3 (city), 4.7 (suburban), and 4.4 (rural) months (Table 3).

**Table 2.** Demographic factors affecting duration of exclusive breastfeeding at six months postpartum in Zhejiang province

Factor	No.	Mean (days)	95% CI	p value
Maternal education (years)				<0.001
≤9	502	64.9	58.9–71.0	
10–12	347	43.4	37.1–49.7	
≥13	574	35.1	30.6–39.7	
Maternal profession				<0.001
Labourer	453	60.7	54.6–66.8	
Office worker	733	38.3	34.1–42.5	
Not working	212	51.0	42.0–60.0	
Maternal age (years)				<0.001
≤24	336	66.0	58.5–73.4	
25–29	757	43.5	39.2–47.9	
≥30	318	38.8	32.3–45.3	
Maternal salary (Yuan)				<0.001
≤3,000	567	61.7	56.2–67.3	
3,000–5,000	431	40.2	34.5–45.8	
≥5,001	396	34.9	29.4–40.3	
Gestation (weeks)				0.069
<37	49	31.9	16.9–46.9	
≥37	1,356	48.2	44.8–51.6	
Birthweight (g)				0.894
≤2,499	26	48.0	24.9–71.1	
2,500–3,999	1,302	47.9	44.5–51.4	
≥4,000	90	45.8	32.5–59.0	
Parity				0.013
Primiparous	1,276	45.5	42.1–48.9	
Multiparous	147	67.3	56.2–78.4	
Delivery method				0.001
Caesarean	969	44.6	40.7–48.5	
Vaginal	457	54.7	48.6–60.7	
Breastfeeding decision made				0.048
Before pregnancy	1,023	50.2	46.2–54.1	
During pregnancy	252	47.8	40.2–55.4	
After birth	146	33.9	24.6–43.2	
Maternal grandmother breastfed				0.812
Yes	1,328	47.8	44.4–51.2	
No	74	43.1	29.2–56.9	
Infant admitted to special-care nursery				0.199
No	1,251	48.8	45.3–52.3	
Yes	146	37.5	27.5–47.5	
Suck time (minute)				<0.001
≤30	452	61.6	55.6–67.6	
>30	910	42.2	38.2–46.2	
Mother attended antenatal classes				<0.001
Yes	920	44.0	40.1–48.0	
No	496	54.0	48.1–59.8	
First feed				0.041
Breastmilk	890	51.4	47.2–55.5	
Other	509	40.4	35.0–45.7	
Baby's gender				0.063
Male	719	45.1	40.6–49.7	
Female	699	50.8	46.0–55.5	
Location				<0.001
City	634	30.0	26.0–34.0	
Suburban	344	64.2	57.3–71.1	
Rural	448	59.2	52.9–65.6	
Back to work				<0.001
Before 6 months	473	39.4	34.3–44.4	
After 6 months	714	60.4	55.5–65.4	

CI=Confidence interval

**Table 3.** Introduction of infant formula in Zhejiang province

Location*	Age when infant formula introduced									
	At discharge		One month		Three months		Six months		More than six months	
	No.	%	No.	%	No.	%	No.	%	No.	%
City	380	67.7	70	12.5	47	8.4	62	11.1	2	0.4
Suburban	123	36.9	42	12.6	20	6.0	145	43.5	3	0.9
Rural	202	39.8	62	12.2	41	8.1	168	33.3	34	6.7

*Pearson χ^2^ statistic 179.3, p<0.001

**Fig. 2.**
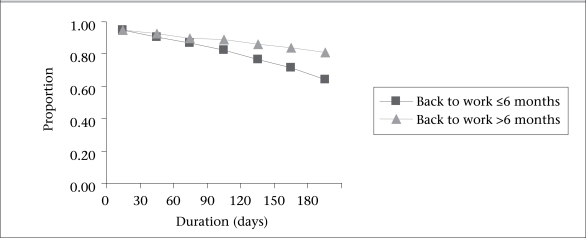
Duration of any breastfeeding up to six months postpartum by status of returning to work

**Fig. 3.**
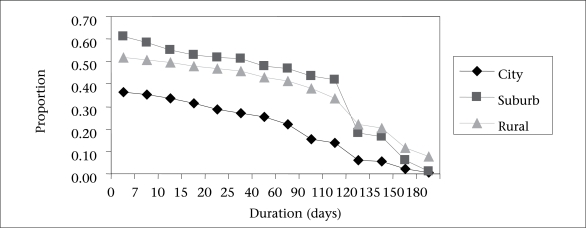
Duration of exclusive breastfeeding up to six months postpartum by location of residence

Table 4 presents the results of the Cox regression analysis for the duration of any breastfeeding. The risk of cessation of any breastfeeding was inversely related to age. The timing of the breastfeeding decision was also a significant factor. The adjusted risk of discontinuing any breastfeeding before six months was higher among mothers who made the breastfeeding decision after pregnancy [hazard ratio (HR)=1.64, 95% confidence interval (CI) 1.10–2.43] than those who made their breastfeeding decision before becoming pregnant. Other significant factors were being admitted to special-care nursery, earlier return of the mother to work (Fig. 2), being given water to drink before one month, early introduction of complementary foods, and the family's place of residence (Fig. 3).

**Table 4.** Cox regression results of significant factors associated with cessation of any breastfeeding in Zhejiang province

Factor	No.	Hazard ratio	95% CI
Maternal age (years)			
≥30	218	1	
25∼29	509	1.710	1.085–2.698
≤24	247	1.520	1.053–2.194
Breastfeeding decision made			
Before pregnancy	689	1	
During pregnancy	175	0.950	0.664–1.360
After Birth	110	1.635	1.099–2.434
Infant admitted to special-care nursery			
No	885	1	
Yes	89	1.508	1.025–2.218
Back to work			
After 6 months	588	1	
Before 6 months	386	1.520	1.129–2.046
Consume water			
After 1 month	632	1	
Within 1 month	342	1.713	1.290–2.274
Introduction of complementary food			
After 3 months	677	1	
Within 3 months	297	1.459	1.074–1.980
Location			
City	368	1	
Suburban	296	0.672	0.466–0.970
Rural	310	0.291	0.183–0.461

CI=Confidence interval

Table 5 presents the results of Cox regression analysis for exclusive breastfeeding. The two significant factors were: mothers who returned to work within six months of delivery and place of residence. Factors associated with the introduction of formula before three months are shown in Table 6. Mothers who made their breastfeeding decision during pregnancy and after the baby's birth were more likely to feed their babies using formula before three months when compared with others who decided before becoming pregnant. Infants whose first feed was not breastmilk were also more likely to be subsequently fed with formula. However, rural mothers were less likely to introduce formula to their babies before three months.

**Table 5.** Cox regression results of significant factors associated with cessation of exclusive breastfeeding in Zhejiang province

Factor	No.	Hazard ratio	95% CI
Back to work			
After 6 months	337	1	
Before 6 months	174	1.471	1.204–1.796
Location			
City	171	1	
Suburban	189	0.739	0.595–0.918
Rural	151	0.590	0.462–0.754

CI=Confidence interval

**Table 6.** Logistic regression results of significant factors associated with infant formula feeding before three months in Zhejiang province

Factor	No.	Odds ratio	95% CI
Breastfeeding decision made			
Before pregnancy	764	1	
During pregnancy	195	1.865	1.150–3.023
After birth	121	3.093	1.814–5.275
First feed			
Breastmilk	643	1	
Other	437	2.177	1.429–3.317
Location			
City	395	1	
Suburban	321	0.833	0.510–1.323
Rural	364	0.417	0.248–0.701

CI=Confidence interval

## DISCUSSION

Following the melamine crisis, the Zhejiang public-health authorities attempted to contact all infants aged 0–3 years—in total, 1.54 million infants. During 2007, of 581,068 reported livebirths, 197,382 (34%) were from parents who migrated from another province (23). The contaminated formulae were inexpensive domestic brands mainly consumed by migrants. For example, 1,229 (73%) of 1,673 infants in a Hangzhou migrant community had been fed with contaminated infant formula at an early age.

The common factors associated with the durations of both exclusive and any breastfeeding were the location of residence of mothers and their early return to work. The infant-feeding practices were different in the rural, suburban and city areas of Zhejiang province (24). The importance of exclusive breastfeeding up to six months of age (and breastfeeding beyond that time) is recognized (25, 26). To support breastfeeding, the Zhejiang Provincial Council has passed regulations to extend maternity leave from six weeks to three months, and employers are requested to ensure that mothers have sufficient time for breastfeeding their infants. Like other countries (27), Chinese mothers should be allowed sufficient time away from work to make feeding arrangements, although employment has less effect in situations where the infant is able to accompany the mother (20).

Another factor that adversely affected the duration of any breastfeeding was the feeding of liquids or food. With the early introduction of complementary foods, the breast receives less stimulation from suckling, resulting in the decrease in the production of milk (28–30). Mothers should be encouraged to delay the introduction of complementary foods until at least six months of age. The influence of complementary foods was consistent with results of previous studies in China (12, 21).

The timing of the breastfeeding decision was known to relate to breastfeeding outcomes in various cultures (10, 31). This was the case in the present study, and it also influenced the early introduction of infant formula. Health professionals should encourage prospective parents to consider breastfeeding at the earliest opportunity and continue to emphasize the advantages of breastfeeding during subsequent antenatal visits.

Most infants (city–99.8%, suburban–99.5%, and rural–92.8%) had consumed or were consuming some infant formula by six months. Education and health-promotion programmes taking the above factors into account to improve the duration of breastfeeding would reduce the risk associated with the consumption of infant formula. The recent contamination episode in China has resulted in the withdrawal of many local infant formula brands from the market. Mothers have few options to feed their infants. They can attempt to relactate, feed international infant formula brands at 3–4 times increased costs. In some instances, they may seek cheaper substitutes, such as cow's milk or even less healthy options (32).

Several limitations must be considered when interpreting the results of the present study. The study sites were restricted to three locations in Zhejiang province based on the financial and logistical support available for this project. While the study locations were selected to be representative of Zhejiang province, further replications at other sites would strengthen the generalizability of the findings. This study was terminated after six months from hospital discharge. A longer follow-up period is recommended in future studies.

In conclusion, this is the first longitudinal cohort study reporting the infant-feeding practices in Zhejiang province of China. The overall rate of any breastfeeding was high but dropped to 74% after six months. Fewer than 5% of the infants reached the WHO and Chinese targets of exclusive breastfeeding until six months of age. The use-rate of infant formula was extremely high (98%), exposing infants to the risk of diseases from contaminated formula. Strategies must be developed to reduce the early introduction of infant formula and to improve the duration of breastfeeding to avoid another melamine disaster in the future.

## ACKNOWLEDGEMENTS

The authors gratefully acknowledge the willing assistance given by the mothers, the hospital staff, nurses, and health workers participating in this study.
